# Aerobic Exercise Regulates Apoptosis through the PI3K/Akt/GSK-3*β* Signaling Pathway to Improve Cognitive Impairment in Alzheimer's Disease Mice

**DOI:** 10.1155/2022/1500710

**Published:** 2022-09-10

**Authors:** Yan Peng, Rui Chi, Gang Liu, Weijie Tian, Jun Zhang, Rihui Zhang

**Affiliations:** College of Kinesiology, Shenyang Sport University, Shenyang, 110102 Liaoning, China

## Abstract

Neuronal apoptosis is an important factor in the etiology of Alzheimer's disease (AD). Aerobic exercise (AE) enhances learning and memory, improves cognitive impairment, increases telomere binding protein expression, and decreases apoptosis regulators, but it remains unclear whether it can improve cognitive impairment caused by neuronal apoptosis in AD. Therefore, this study investigated whether an 8-week running table exercise intervention could reduce apoptosis and improve cognitive function in the hippocampal neurons of AD model mice. After the exercise intervention, we evaluated the learning memory ability (positioning, navigation, and spatial search) of mice using a Morris water labyrinth, Nissl staining, immunohistochemistry, and protein application to detect hippocampal PI3K/Akt/GSK-3*β* signaling pathway protein and hippocampal neuronal cell apoptosis protein B cell lymphoma 2 (Bcl-2) and apoptosis-promoting protein bcl-2-related X (Bax) protein expression. The results showed that aerobic exercise improved the location and spatial exploration ability of mice, increased the number of PI3K- and p-Akt-positive cells, increased the expression of PI3K, p-Akt, and bcl-2 proteins, decreased the expression of GSK-3*β* and Bax proteins, and increased the bcl-2/Bax ratio of mice. The results suggest that aerobic exercise can reduce apoptosis and improve cognitive function in AD mice. The molecular mechanism may involve activation of the PI3K/Akt/GSK-3*β* signaling pathway.

## 1. Introduction

Alzheimer's disease (AD) is a neurodegenerative disorder characterized by senile plaques (SP) formed by *β*-amyloid (A*β*) deposition and neurogenic fibrillary tangles (NFT) formed by tau protein hyperphosphorylation [1, 2], as well as by massive neuronal loss [3, 4]. AD is a multifactorial disease whose specific pathogenesis is not clear. The existing theories include A*β* deposition, excessive tau protein phosphorylation, free radical damage, choline deficiency, reactive synapse loss, and neurodegeneration [5, 6]. The occurrence of AD eventually leads to apoptosis of nerve cells [7]. AD is marked by cognitive decline [8] and a progressive decline in independence [9, 10]. The incidence of AD is increasing with the aging of the population, and its high mortality rate is mainly due to the complex and unclear etiology and limited intervention options. Therefore, the prevention and treatment of AD have become a global concern.

Phosphatidylinositol 3-kinase (PI3K)/protein kinase B (Akt) is a regulator of cell proliferation [11], growth, and survival, inhibiting many neurotoxins and reducing neuronal apoptosis [12]. Its downstream molecule is GSK3. GSK3 includes GSK3*α* and GSK3-*β*, of which GSK3-*β* is considered a key participant in AD pathophysiology, and imbalances in the kinase affect the hyperphosphorylation of tau protein, cognitive impairment, neurogenesis, synaptic function, and apoptosis induced by A*β* over deposition [13, 14]. A large number of apoptotic neurons were found in the cerebral cortex and hippocampus in the early stages of AD [15]. The PI3K/Akt/GSK3-*β* signaling pathway plays an important transduction role in neuronal apoptosis in AD [16], and its downstream apoptosis homeostasis protein Bcl-2/Bax is used as a key determinant of apoptosis [17].

Aerobic exercise benefits brain growth and development and increases hippocampal volume, delays decline, reverses pathology, and prevents the development of AD by reducing oxidative stress, apoptosis, neuroinflammation, and mitochondrial dysfunction; it also promotes growth factors and improves memory [18, 19]. Exercise improves learning and memory capacity in AD through long-term potentiation (LTP) and stimulation of the body's production of neurotrophic factors (e.g., BDNF) and nerve growth factors [20, 21].

Therefore, this study used the intraperitoneal injection of D-galactose and aluminum trichloride for AD modeling, employing the concept of early prevention. Three-month-old mice with AD were given 8 weeks of aerobic exercise at the same time as interventions (early detection and intervention with exercise intervention at the same time as modeling). The Morris water maze (MWM) was used to test the learning and memory ability of mice, Nissl staining and immunohistochemistry were used to detect the number of positive cells, and Western blot was used to measure the protein expression of each molecule to investigate whether 8 weeks of the running table exercise intervention would affect the PI3K/Akt/GSK3-*β* signaling pathway in AD mice and whether it could improve cognitive function by reducing hippocampal neuronal apoptosis. We sought to provide a theoretical and experimental basis for elucidating the effect of aerobic exercise on delaying the onset of AD.

## 2. Materials and Methods

### 2.1. Ethical Approval

The mice were purchased from Spelford (Beijing, China) Biotechnology Co. Ltd. (mice production license no. 11401500058072). They were housed in the research building of the rehabilitation center of Liaoning University of Traditional Chinese Medicine (Shenyang, China), and all operations of this experiment conformed to the ethical standards of animal experiments of the rehabilitation center of Liaoning University of Traditional Chinese Medicine.

### 2.2. Animals

Sixty-four three-month-old healthy C57BL/6J mice weighing 27 ± 3 g were housed three mice per cage. The temperature was maintained between 18°C and 22°C, the humidity was 45% to 55%, good ventilation was maintained, and the mice were fed and watered freely. Mice were randomly grouped into 4 groups of 16 mice each: control (C) (*n* = 16), exercise control (EC) (*n* = 16), AD model (M) (*n* = 16), and exercise model (EM) (*n* = 16). Groups C and EC were intraperitoneally injected with normal saline every day, while groups M and EM were intraperitoneally injected with D-galactose (dose: 80 mg/kg/day) and aluminum chloride (dose: 5 mg/kg/day) for 8 consecutive weeks. Modeling was done in the morning, and the running platform was used in the afternoon. During modeling, the EM and EC groups were subjected to treadmill exercise for 8 consecutive weeks. Eight mice in each group were used for immunohistochemistry, and the other 8 mice were used for Western blotting.

### 2.3. Movement Program

The exercise control and exercise model groups first performed a 3-day acclimatization running exercise for 20 min/day at a speed of 8–10 m/min and rested for 1 day after the acclimatization exercise, while the EC and EM groups performed formal exercise for 8 weeks, 5 times per week, with the exercise speed increasing from 12 m/min to 15 m/min and the time increasing from 15 min to 45 min, with an incline of 0°.

### 2.4. Behavioral Tests in Mice

The MWM is a classical experiment to test the cognitive function of mice. The experimental setup consists of a computer system, a dongle, a camera device, a circular pool (80 cm diameter, 33 cm high, and white interior), and a movable platform (15 cm high, 7 cm diameter, and white). The computer system divided the circular pool equally into four quadrants through the camera system, placed the platform in the first quadrant, and placed the mice in the fourth quadrant, keeping the platform position constant throughout the experiment without any change during the experiment. The learning memory ability of the four groups of experimental mice was observed through orientation navigation and spatial exploration experiments.

#### 2.4.1. Positioning Navigation Experiment

The mice were placed into the water from the wall of the pool marked in the fourth quadrant in turn, and the time taken by the mice to find the platform within 120 s was recorded as the escape latency; if the mice did not find the hidden platform in the water for more than 120 s, the escape latency was recorded as 120 s. The escape latency of the mice was calculated for 5 days.

#### 2.4.2. Spatial Exploration Experiment

The platform was removed on day 6, the mice were placed in the water from the fourth quadrant, and the number of platform crossings within 120 s was recorded.

### 2.5. Changes in the Body Weight of Mice

Before the experiment, the body weight of the mice in each group was basically the same, without a significant difference. After 8 weeks of continuous injection of D-galactose and aluminum chloride, the body weight of mice in group M decreased significantly compared with group C (*P* < 0.01) and the body weight of mice in group EM increased significantly compared with group M (*P* < 0.01).


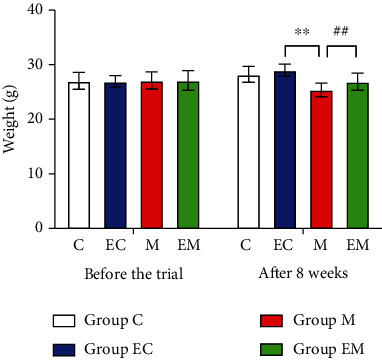


### 2.6. Nissl Staining

Forty-eight hours after the end of the behavioral assay, mice were anesthetized with 0.3% sodium pentobarbital 30 mg/kg by intraperitoneal injection, the brains were severed, and hippocampal tissues were rapidly separated and put into liquid nitrogen for freezing. Paraffin section preparation consisted of dehydration, transparency, embedding, slicing, baking, xylene alcohol dewaxing, distilled water soaking for 5 min, placing in Nissl staining solution and staining for 5 min, distilled water washing twice for several seconds each time, 95% ethanol fractionation for 1 min, anhydrous ethanol for 5 min, anhydrous ethanol for 5 min, sealing with neutral gum, and microscopic observation of nisin vesicles and nuclei in violet-blue color on fully solidified sections.

### 2.7. Immunohistochemical Experiments

The first two steps were the same as those for the Nissl staining. These steps were followed by a 5 min rinse in running water at the end of dewaxing, citrate repair, and a 16 min incubation with an endogenous peroxidase blocker. Afterwards, the samples were washed with PBS and we added the primary antibody (PI3Kp110, p-Akt, and GSK-3*β*, all 1 : 50 rabbit anti-mouse donor antibody) dropwise. The samples were incubated at 4°C overnight, washed 5x in PBS, and then incubated with the universal secondary antibody (1 : 200) for 25 min. This was followed with another PBS wash and diaminobenzidine (DAB) for color development. Color development was terminated by adding ddH_2_O after 5–8 min, the samples were restained using hematoxylin and dehydrated with xylene alcohol, and the films were sealed. Microscopy was performed, three sections were measured for each specimen, and five fields were randomly selected in the CA1 region of each section. The ImageJ 8.0 image analysis system was used to calculate the mean of the positive cell counts.

### 2.8. Western Blotting

The hippocampal tissues were weighed. Tissues of the same weight were placed into a glass homogenizer precooled with ice, and lysate and protease inhibitor (ratio 100 : 1) were added. Grinding was performed in an ice bath environment; after standing, centrifuge was placed at 4°C and centrifuged at 12000 r/min for 15 min. The supernatant was aspirated, and the concentration of its protein was determined by BCA. A protein loading buffer (5x) was added, and the protein was denatured for 5 min. SDS-polyacrylamide gel electrophoresis (SDS-PAGE) separation was then performed. We performed the electrophoretic transfer of proteins to polyvinylidene difluoride membrane (PVDF). Then, 5% skim milk powder was added the shaker for 1 h and the following primary antibodies were added and kept overnight at 4°C: rabbit anti-PI3K p110 (1 : 500), mouse anti-Akt (1 : 2000, Proteintech, Wuhan, China), mouse anti-phospho-Akt Ser473 (1 : 2000, Proteintech, Wuhan, China), rabbit anti-GSK-3*β* (1 : 1000, Proteintech, Wuhan, China), rabbit anti-Bax (1 : 1000, Boster, Wuhan, China), and rabbit anti-Bcl-2 (1 : 1000, Boster, Wuhan, China). The TBS + Tween wash buffer was used three times, and the corresponding secondary antibody (1 : 5000, Proteintech, Wuhan, China) was incubated for 1 h at room temperature and washed well with TBST. *β*-Actin was used as an internal reference, enhanced chemiluminescent solution (ECL) (Beyotime, China) was used for color development, and the ratio of target protein optical density values to *β*-actin optical density was counted and analyzed.

### 2.9. Statistical Analysis

Data were analyzed using ImageJ 8.0 and SPSS 19.0 software. GraphPad Prism software was used for plotting. Each experiment was repeated five times. The data obtained were expressed as the mean ± standard deviation, and one-way analysis of variance (ANOVA) was used for comparison between groups. Statistical significance was considered at *P* < 0.05.

## 3. Results

### 3.1. Aerobic Exercise Improves Learning and Memory in AD Mice

#### 3.1.1. Experimental Results for Positioning Navigation

**Figure 1:**
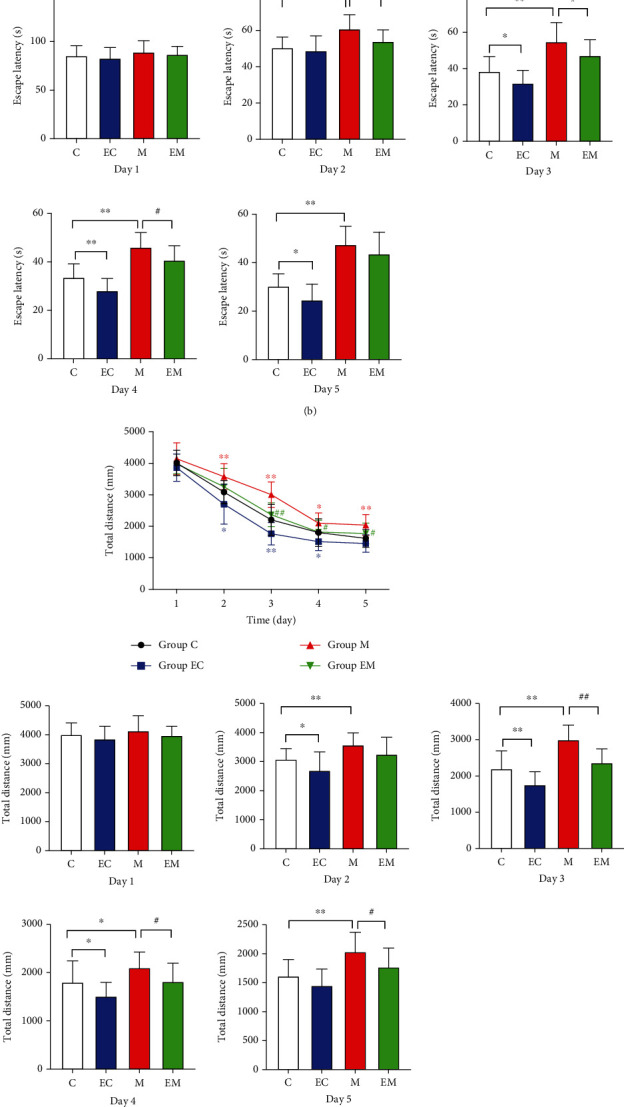
(a) Morris water maze swimming path for each group of mice. (b) Evasion latency time of mice. (c) Evasion latency time of mice. (d) Number of times that mice crossed the platform. ^∗^*P* < 0.05 and ^∗∗^*P* < 0.01 vs. group C; ^#^*P* < 0.05 and ^##^*P* < 0.01 vs. group M.

Figure 1(a) shows the water maze swimming path for the MWM positioning navigation experiment, and Figure 1(b) shows the escape latency time. On the first (Figure 1(b1)), third, fourth, and fifth days of the experiment (Figures 1(b3), 1(b4), and 1(b5), respectively), the change was obvious. The time taken to escape in group M was significantly higher than that in group C. Group M spent more time looking for hidden platforms in the water, and on days 2, 3, and 4 (Figures 1(b2), 1(b3), and 1(b4), respectively), the time spent by group EM decreased significantly compared with group M.  Figure 1(c) shows the escape distance, and on days 1, 2, 3, 4, and 5 (Figures 1(c1), 1(c2), 1(c3), 1(c4), and 1(c5), respectively), the evasion distance of group M was significantly longer than that of group C. On days 2, 3, and 4, the evasion distance of mice in group EC was lower than that of group C. On days 3, 4, and 5, the evasion distance of group EM decreased significantly compared to that of group M.

Figure 1(d) shows the number of times that mice traversed the platform to detect the spatial exploration ability of mice. Compared with group C, the number of times that mice traversed the platform was significantly reduced in group M (*P* < 0.01). After 8 weeks of AE, the number of times that mice traversed the platform was significantly increased in group EM compared with group M (*P* < 0.05). These data suggest that the learning and memory abilities of AD mice were significantly decreased, and after 8 weeks of running platform exercise, their positioning navigation and spatial exploration ability was improved and their cognitive function was significantly improved.

### 3.2. Aerobic Exercise Attenuates Neuropathic Damage in the Hippocampus of AD Mice

**Figure 2:**
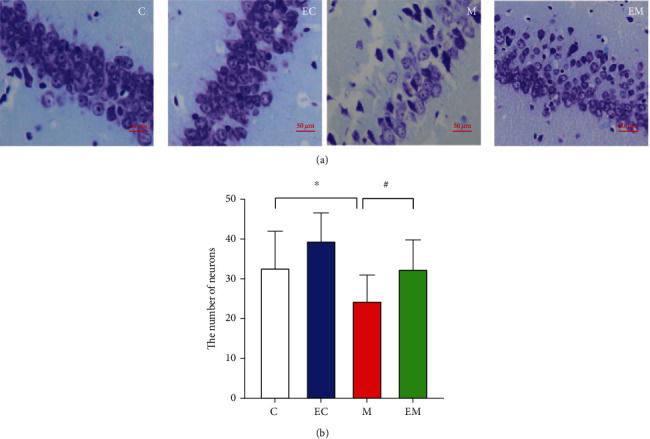
Effect of exercise on apoptosis in the hippocampus of AD mice. (a) Nissl staining of the hippocampal CA1 region. (b) The number of positive cells in the hippocampal CA1 region. Results are mean ± SD (*n* = 8/group). ^∗^*P* < 0.05 and ^∗∗^*P* < 0.01 vs. group C; ^#^*P* < 0.05 and ^##^*P* < 0.01 vs. group M. Scale bar: 50 *μ*m.

Nissl staining of the mouse hippocampal CA1 region was performed, and the results are shown in Figure 2. Cells in groups C and EC were regularly and closely arranged, with large and round nuclei and abundant numbers of Nissl vesicles in the cytoplasm. In group M, Nissl vesicles were heavily lysed and disappeared, cell gaps increased, and Nissl vesicles in the cytoplasm were reduced. Compared with group M, the neurons in group C had a clear structure and evenly distributed Nissl vesicles in the cytoplasm. After 8 weeks of aerobic exercise, the EM group showed improvement compared to the M group and gradually approached the normal condition. The number of neurons in group M was significantly reduced compared to group C. After 8 weeks of aerobic exercise, the number of neurons in group EM was significantly increased compared to group M. The results suggest that exercise can reduce hippocampal neuronal cell injury.

### 3.3. Exercise Exerts Neuroprotective Effects through Modulation of the PI3K/Akt/GSK-3*β* Signaling Pathway

#### 3.3.1. Immunohistochemical Staining Results

**Figure 3:**
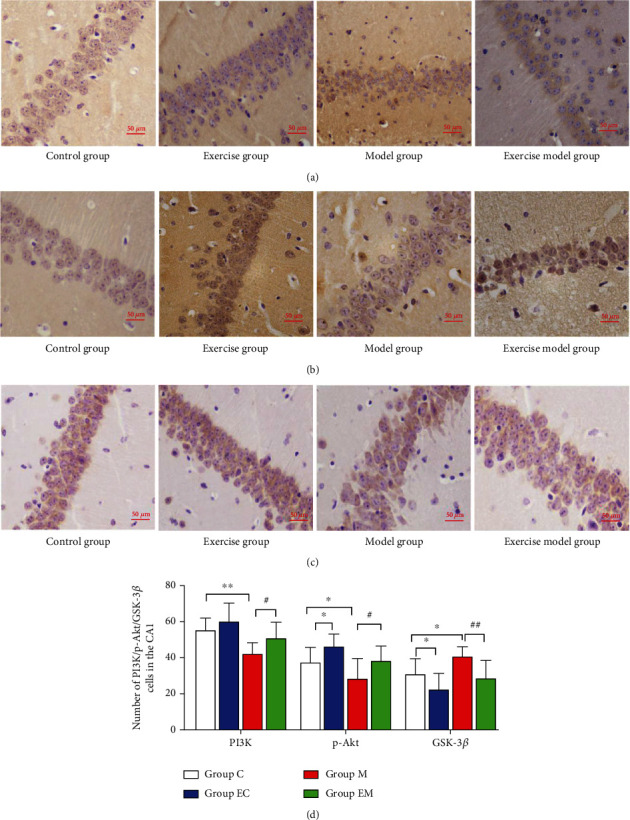
Number of PI3K-, p-Akt-, and GSK-3*β*-positive neurons in hippocampal CA1 of each group of mice. (a–c) PI3K, P-Akt, and GSK-3*β* immunohistochemical results of the hippocampal CA1 region in each group. (d) The number of immunohistochemical-positive cells in the hippocampal CA1 area of each group. Results are mean ± SD (*n* = 8/group). ^∗^*P* < 0.05 and ^∗∗^*P* < 0.01 vs. group C; ^#^*P* < 0.05 and ^##^*P* < 0.01 vs. group M. Scale bar: 50 *μ*m.

As shown in Figure 3, PI3K, p-Akt, and GSK-3*β* were expressed in the hippocampal tissues of all groups and the positive particles were mainly expressed in the cell membrane, cytoplasm, and cytoplasmic sites in the CA1 area. Compared to group C, the number of p-Akt- and GSK-3*β*-positive cells in the EC group was significantly increased, the number of GSK-3*β* and p-Akt positive cells in groups EC and M was reduced, and PI3K was highly significantly increased. Compared to group M, the number of PI3K- and p-Akt-positive cells in group EM was increased and GSK-3*β* was highly significantly decreased.

### 3.4. Western Blot Results

**Figure 4:**
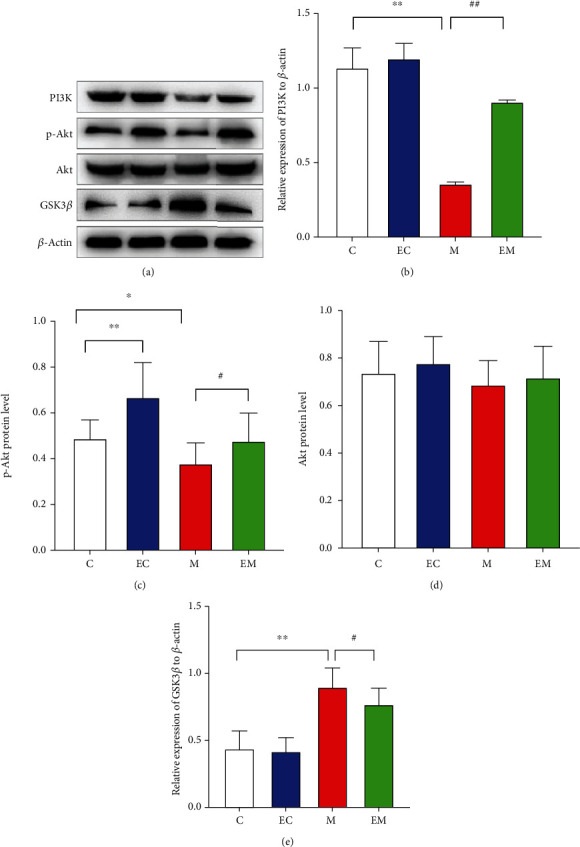
Hippocampal PI3K, p-Akt, Akt, and GSK-3*β* protein expression in various groups of mice. (a) PI3K, p-Akt, Akt, and GSK-3*β* Western blot images. (b–e) PI3K, p-Akt, Akt, and GSK-3*β* expression levels in each group of mice. *β*-Actin was used as an internal reference protein, and the data are mean ± SD (*n* = 8/group). ^∗^*P* < 0.05 and ^∗∗^*P* < 0.01 vs. group C; ^#^*P* < 0.05 and ^##^*P* < 0.01 vs. group M.

As shown in Figure 4, there was no significant difference in Akt between the groups and exercise increased PI3K and p-Akt expression and decreased GSK-3*β* levels. The findings suggest that the neuroprotective effects of exercise and the improvement of cognitive function may be related to the regulation of the PI3K/Akt/GSK-3*β* pathway.

### 3.5. Aerobic Exercise Reduces Hippocampal Neuronal Apoptosis

**Figure 5:**
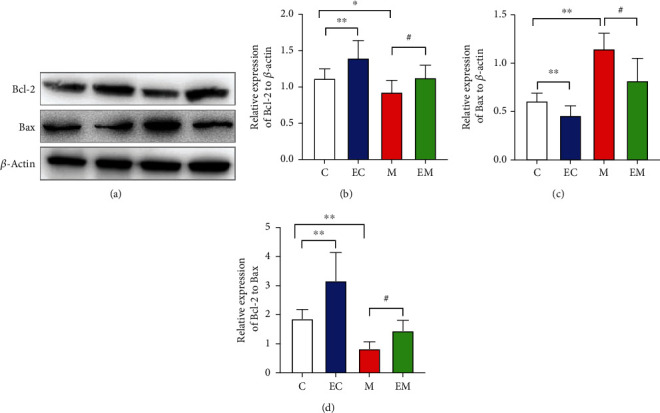
Expression of apoptotic proteins Bcl-2, Bax, and Bcl-2/Bax in the hippocampus of various groups of mice. (a) Western blot analysis, Bcl-2, and Bax images. (b–d) Bcl-2, Bax protein expression, and Bcl-2/Bax levels in each group of mice. *β*-Actin was used as an internal reference protein, and the data are mean ± SD (*n* = 8/group). ^∗^*P* < 0.05 and ^∗∗^*P* < 0.01 vs. group C; ^#^*P* < 0.05 and ^##^*P* < 0.01 vs. group M.

Apoptosis is a key pathological contributor to neurodegenerative diseases like AD [22]. To be specific, the Bcl-2 protein family is one of the most critical apoptotic families. As shown in the Western blot results in Figure 5, Bcl-2 expression and the Bcl-2/Bax ratio were increased in group EC compared with group C and the opposite was true for group M. After 8 weeks of AE, Bcl-2 expression and the Bcl-2/Bax ratio were significantly increased and Bax levels were decreased in group EM compared with group M. The results suggest that exercise may activate the PI3K/Akt/GSK-3*β* pathway, adjust the balance between Bcl-2 and Bax, and reduce the excessive apoptosis of cells.

## 4. Discussion

In this study, we examined the learning, memory ability, and hippocampal apoptosis in AD mice. Compared with group C, the escape latency and escape distance of mice in group M increased and the number of platform crossings decreased. By successfully establishing a mouse model of AD, we explored the mechanism of the role of aerobic exercise in delaying AD. The results showed that aerobic exercise improved the learning memory ability of AD mice through a molecular mechanism whereby aerobic exercise slows apoptosis in AD by activating the PI3K/Akt/GSK-3*β* signaling pathway and increasing the Bcl-2/Bax ratio in the hippocampus.

In this study, detection of cognitive function in mice by MWM showed that AE improved learning memory capacity and reduced neuronal damage in AD mice and positioning navigation and spatial exploration ability was significantly decreased in the model group. After 8 weeks of aerobic exercise, the evasion latency time gradually decreased, the number of platform crossings increased, and the cognitive function improved. AD also caused a decrease in the number of hippocampal Nissl-positive cells in mice, and the number of hippocampal neurons increased significantly after 8 weeks of aerobic exercise. Exercise promotes structural changes in the brain [23], promotes an increase in hippocampal and prefrontal cortex cells [24], enhances brain plasticity [25, 26], blocks glial activation [27], promotes hippocampal neurogenesis [28], and improves learning and memory abilities [29].

The development of a good exercise program with appropriate exercise frequency, duration, and intensity will have a better effect on the cognitive function and physical function of the elderly [30, 31]. Lu et al. [32] conducted aerobic exercise in AD rats for 4 weeks and found that exercise significantly alleviated the neurodegeneration in the hippocampal CA1 region of rats and improved the cognitive function of mice. Similarly, in a study that conducted 4 weeks of running table exercise in rats with AD induced by A*β*1-42 injection in the hippocampus, exercise accelerated the clearance rate of central and peripheral A*β* and improved the learning memory ability of AD rats [33, 34]. Exercise improves patients' cognitive function, eases the burden on family members, and continuously improves patients' quality of life [35].

GSK-3*β* is a major kinase in brain tissue that is mainly involved in brain cell survival and apoptosis [36]. GSK-3*β* protein is a direct substrate of PI3K/Akt [37], which plays an essential role in the regulation of AD, and the adjustment of this pathway can reduce apoptosis and delay the development of AD, which may be one of the factors that improve cognitive function in terms of the molecular biological mechanism. In this study, the PI3K/Akt/GSK3-*β* pathway was examined by immunohistochemistry and Western blot and the number and protein contents of PI3K- and p-Akt-positive cells in the hippocampus were decreased, while the GSK-3*β* content was increased, in group M mice. Eight weeks of running table exercise increased the number and level of PI3K- and p-Akt positive cells and decreased GSK-3*β*-positive neuronal number and protein expression. Exercise exerts neuroprotective effects via the PI3K/Akt pathway [38] and suppresses GSK3*β* expression while improving hippocampal morphology and learning memory in mice with AD [39], which may explain the protection of neurons in AD mice by AE through the PI3K/Akt/GSK3-*β* pathway.

Apoptosis of neuronal cells eventually leads to the development of AD, and the damage to the body from the loss of neurons increases with age. The Bcl-2 protein family is a family of downstream proteins in all apoptotic pathways [40, 41] and includes Bcl-2 and Bax, with antiapoptotic molecule Bcl-2 and apoptosis-promoting molecule Bax being a set of apoptotic steady-state factors [42]. Bcl-2 is a key factor that inhibits apoptosis [43, 44]; it is highly expressed in the normal brain and protects cells from death to some extent. The Bcl-2/Bax ratio usually determines apoptosis. AE plays a neuroprotective role by inhibiting neuronal apoptosis in the brain [45] and regulating neuronal production and apoptosis by modulating cell growth factors [46]. AE also increases telomere-binding protein expression and decreases apoptosis regulatory factors, thus preventing cellular senescence [47]. In a previous study, exercise increased Bcl-2 and decreased Bax levels and inhibited apoptosis in the mouse hippocampus, possibly through activation of the PI3K/Akt/GSK-3*β* pathway. A study of AD mice subjected to 4 weeks of strength training found that exercise decreased the Bax/Bcl-2 ratio in the hippocampus of mice [48]. A study on 6 weeks of aerobic exercise in AD rats found that exercise increased Bcl-2, decreased Bax content, increased the Bcl-2/Bax ratio, and inhibited apoptosis in AD [49].

Whether exercise regulates the development of AD by modulating the PI3K/Akt/GSK-3*β* pathway has not been elucidated. In this study, we preliminarily verified that exercise may enhance learning memory and attenuate hippocampal neuronal apoptosis in AD by activating the PI3K/Akt/GSK-3*β* pathway. These results suggest that exercise can protect neurons by inhibiting their apoptosis and increasing synaptic plasticity.

In summary, this study successfully constructed an AD mouse model. We confirmed that 8 weeks of AE could improve cognitive function and enhance learning and memory and that aerobic exercise could attenuate hippocampal apoptosis in AD mice by modulating the PI3K/Akt/GSK-3*β* pathway. We confirmed that an exercise intervention may have certain preventive and therapeutic effects on the development of AD. In the future, we will perform more studies to fully demonstrate the feasibility of early exercise intervention in the development of AD.

## 5. Conclusion

Our results show that aerobic exercise improves learning and memory and inhibits apoptosis in hippocampal cells, possibly due to activation of the PI3K/Akt/GSK-3*β* pathway, which regulates a series of downstream protein-associated responses.

## Data Availability

In this paper, we include data that support the results of this study.
